# Macrotrabecular-Massive Subtype Is Associated with a High Risk of the Recurrence of Hepatocellular Carcinoma

**DOI:** 10.3390/jcm15020502

**Published:** 2026-01-08

**Authors:** Sung Hwan Yoo, Ji Hae Nahm, Hye Young Chang, Jung Il Lee, Jin Hong Lim, Hyun Woong Lee

**Affiliations:** 1 Department of Internal Medicine, Gangnam Severance Hospital, Yonsei University College of Medicine, Seoul 06273, Republic of Korea; oscar0125@yuhs.ac (S.H.Y.);; 2 Department of Pathology, Gangnam Severance Hospital, Yonsei University College of Medicine, Seoul 06273, Republic of Korea; 3 Department of Surgery, Gangnam Severance Hospital, Yonsei University College of Medicine, Seoul 06273, Republic of Korea

**Keywords:** hepatocellular carcinoma, macrotrabecular-massive subtype, recurrence, prognosis, histopathology, immune microenvironment, survival

## Abstract

**Background**: Hepatocellular carcinoma (HCC) is one of the leading causes of cancer-related mortality worldwide, mainly due to its high recurrence rate after curative treatments. The macrotrabecular-massive (MTM) subtype has recently been recognized as an aggressive histologic variant associated with vascular invasion and poor differentiation. This study aimed to investigate the prognostic impact of the MTM subtype on recurrence after surgical resection of HCC. **Methods**: We retrospectively reviewed 171 patients who underwent curative hepatic resection for HCC between January 2007 and December 2017 at Gangnam Severance Hospital, Seoul, Korea. Clinicopathologic parameters, including immune-related features such as lymphoid infiltration and tertiary lymphoid structures (TLSs), were evaluated. Recurrence-free survival (RFS) and overall survival (OS) were analyzed using Kaplan–Meier and Cox regression analyses. **Results**: During a median follow-up of 4.4 ± 3.4 years, 74 patients (43.3%) experienced recurrence. The MTM subtype was significantly more frequent in the recurrence group than in the non-recurrence group (47.3% vs. 21.6%, *p* < 0.001). Multivariate analysis identified MTM subtype as an independent predictor of recurrence (hazard ratio, 1.88; 95% CI, 1.14–3.10; *p* = 0.013). Lymphoid infiltration and TLSs were not associated with prognosis. Kaplan–Meier analysis showed higher recurrence in MTM-positive cases (*p* = 0.001), whereas OS did not differ significantly (*p* = 0.094). **Conclusions**: The macrotrabecular-massive subtype is an independent histopathologic predictor of recurrence after curative resection in HCC. Incorporating MTM subtype recognition into postoperative risk assessment may enhance patient stratification and inform future adjuvant therapy strategies.

## 1. Background

Hepatocellular carcinoma (HCC) is the fifth most commonly diagnosed cancer globally and the second leading cause of cancer-related mortality worldwide [1,2]. Hepatocellular carcinoma (HCC), the most prevalent primary liver malignancy, is primarily driven by risk factors such as hepatitis B virus (HBV) or hepatitis C virus (HCV) infection, excessive alcohol consumption, and metabolic syndrome related to diabetes and obesity [2,3]. Despite advancements in diagnostic modalities and therapeutic strategies, the prognosis of HCC remains poor, primarily due to its high recurrence rate following curative treatments such as surgical resection, radiofrequency ablation (RFA) or liver transplantation [3]. In the past decade, advancements in high-throughput molecular technologies have enabled a deeper understanding of the key molecular mechanisms driving the development and progression of hepatocellular carcinoma (HCC). This cancer is now recognized as a highly heterogeneous disease, comprising distinct transcriptomic subgroups characterized by diverse genetic alterations [4,5,6,7,8,9].

As such, identifying histopathological and molecular features associated with recurrence risk is essential for improving patient outcomes and tailoring therapeutic approaches.

Recent studies have highlighted the heterogeneity of HCC at both the histological and molecular levels. Among the various subtypes, the macrotrabecular-massive (MTM) subtype has emerged as a distinct histological variant characterized by its large, trabecular growth pattern and aggressive clinical behavior. This subtype has been linked to a higher prevalence of vascular invasion, poor differentiation, and unfavorable outcomes, suggesting its potential role as a prognostic marker.

In this study, we aim to explore the association between the MTM subtype and the recurrence risk of HCC. By investigating clinicopathological data and recurrence patterns, we seek to provide insights into the prognostic significance of the MTM subtype and its implications for personalized management strategies in HCC patients.

## 2. Methods

### 2.1. Study Population

This retrospective cohort study was performed with 171 hepatocellular carcinoma (HCC) patients who were histopathologically diagnosed after hepatic resection in a tertiary hospital, Gangnam Severance Hospital, located in Seoul, Korea. recorded between January 2007 and December 2017. Curative hepatic resection was defined as complete macroscopic tumor removal with negative surgical margins.

Especially, the prognostic significance of lymphocyte infiltration and macrotrabecular-massive subtype in a series of 171 patients with HCC treated by surgical resection were analyzed by pathologist.

The exclusion criteria were as follows: (1) HCC patients who underwent any kind of prior treatment before hepatic resection, (2) patients with multiple HCCs, (3) extrahepatic metastasis at the time of surgery, (4) HCC patients without pathology slides available for review, (5) liver transplantation status after hepatic resection, (6) patients with comorbidities such as congestive heart failure, renal failure, and other malignancies that could affect the overall survival of HCC patients, and (7) follow-up period less than 6 months.

The study protocol was performed in accordance with the principles of the 1975 Declaration of Helsinki, and approved by the Yonsei University Gangnam Severance Hospital, Institutional Review Board (3-2021-0255). The need for informed consent was waived by the ethics committee/Institutional Review Board of Yonsei University Gangnam Severance Hospital, because of the retrospective nature of the study.

### 2.2. Baseline Characteristics of Patients Treated by Surgical Resection

Clinical information and laboratory data of 171 patients treated by surgical resection were retrospectively studied from electronic medical records, including patients’ demographics such as age, gender at surgery and survival data; underlying disease such as hypertension, diabetes mellitus; presence of liver cirrhosis; etiology of chronic liver disease; levels of alpha-fetoprotein (AFP) and protein induced by vitamin K absence (PIVKA-II), Child–Pugh class.

### 2.3. Characteristics of Tumor and Stage Work Up

In addition to individual factors, cancer-related variables such as maximal tumor size, tumor number, portal vein invasion, and bile duct invasion and precise caner stage like Barcelona Clinic Liver Cancer (BCLC) stage, American Joint Committee on Cancer (AJCC) 8th stage were also investigated.

### 2.4. Pathologic Analysis by Pathologist

For each case, all available histological slides were reviewed. Especially, lymphoid cell infiltration [10,11]; tertiary lymphoid structures (TLSs) [12,13]; tumor infiltrating lymphocyte [14]; macrotrabecular-massive subtype [15], and Edmonson-Steiner grade were reviewed by the pathologist working in Gangnam Severance Hospital.

Lymphoid cell infiltration is a common feature of many human tumors, and the degree of infiltration has been considered to be a measure of the host immune response [11]. TLSs, a concept that has emerged recently, could contribute to intratumoral immune responses and sustains B cell maturation and antibody production that is associated with anti-tumor effects [12,16]. Tumor infiltrating lymphocytes are also known to play a pivotal role in mediating response to chemotherapy and improving clinical outcomes. MTM-HCC subtype was defined by the presence of a predominant (>50%) macrotrabecular architecture (more than six cells thick) [17]. Pathological diagnosis and grading were also made according to Edmondson-Steiner grading system [18,19].

### 2.5. Definition of Early and Late Recurrence

Tumor recurrence was defined as the radiologic or histologic detection of new HCC lesions during follow-up. The recurrence-free interval was calculated from the date of hepatic resection to the date of first documented recurrence and expressed in months.

Based on prior studies demonstrating distinct biological mechanisms underlying early versus late recurrence, postoperative recurrence occurring within 24 months was classified as early recurrence, whereas recurrence occurring after 24 months was considered late recurrence. Patients without recurrence were censored at the time of last follow-up. Cumulative incidence of tumor recurrence was estimated using a competing risk approach, treating death without recurrence as a competing event.

### 2.6. Statistical Analysis

Patients’ baseline characteristics were expressed as mean with standard deviation in the case of continuous variables and numbers with percentages in the case of categorical variables.

Risk factors for the recurrence of hepatocellular carcinoma were investigated using Cox regression analysis. The cumulative incidence rates of tumor recurrence and overall survival were investigated using the Kaplan–Meier method and compared by the log-rank test. Subsequently, cumulative incidence of tumor recurrence and survival rates according to macrotrabecular-massive subtype positive were analyzed.

For patients who developed recurrence, clinicopathologic variables were compared between the early and late recurrence groups. Categorical variables were analyzed using the χ^2^ test or Fisher’s exact test, and continuous variables using Student’s *t*-test or the Mann–Whitney U test, as appropriate.

To identify factors associated with early recurrence, logistic regression analysis was performed with early (vs. late) recurrence as the dependent variable. Variables with *p* < 0.10 in the univariate analysis, together with clinically relevant factors, were included in the multivariable model. The association between MTM subtype and early recurrence was a prespecified analysis based on prior evidence of its aggressive biology.

All missing values were not used for analysis. All statistical analyses were performed using SPSS version 25.0 (IBM Co., Armonk, NY, USA) and R software (version 4.3.1; R Foundation for Statistical Computing, Vienna, Austria), including Kaplan–Meier survival analysis, Cox regression modeling, and logistic regression for early versus late recurrence.

## 3. Results

### 3.1. Baseline Characteristics

A total of 171 patients with HCC treated by surgical resection between January 2007 and December 2017 were retrospectively analyzed. Median follow-up duration was 4.4 ± 3.4 years (3.2 months–15.5 years). Baseline characteristics of patients with hepatocellular carcinoma treated by surgical resection are summarized in Table 1.

A total of 171 patients were included in the study, with 74 experiencing recurrence and 97 showing no recurrence after surgical resection. The mean age was significantly higher in the recurrence group compared to the no-recurrence group (65.1 ± 8.6 vs. 61.6 ± 10.8 years, *p* = 0.021). However, there were no statistically significant differences between the two groups in terms of sex distribution, BMI, etiology (including HBV, HCV, alcohol, MASH, and unknown causes), diabetes, hypertension, or liver cirrhosis. Serological tumor markers AFP and PIVKA-II did not show significant differences between the groups. Similarly, the Child–Pugh classification was comparable across groups, with most patients in class A. Among tumor characteristics, maximal tumor size was significantly larger in the recurrence group than in the no-recurrence group (4.3 ± 3.2 vs. 3.4 ± 2.5 cm, *p* = 0.032). Tumor number, resection margin positivity, and vascular/biliary invasions (including microvascular, portal vein, and bile duct invasions) were not significantly different between the groups.

The distribution of clinical stages based on both the BCLC and AJCC 8th edition staging systems showed no significant differences between the recurrence and no-recurrence groups (*p* = 0.230 and *p* = 0.244, respectively). The majority of patients in both groups were classified as BCLC stage A and AJCC stage IB.

Immune-related features, including lymphoid cell infiltration, tertiary lymphoid structure presence, and tumor-infiltrating lymphocytes, also did not differ significantly between the groups. However, the macrotrabecular-massive subtype was significantly more common in the recurrence group than in the no-recurrence group (47.3% vs. 21.6%, *p* < 0.001), suggesting its potential association with recurrence risk. Edmonson grade and follow-up duration were similar between the groups, with no statistically significant differences observed.

### 3.2. Recurrence and Survival

**Figure 1:**
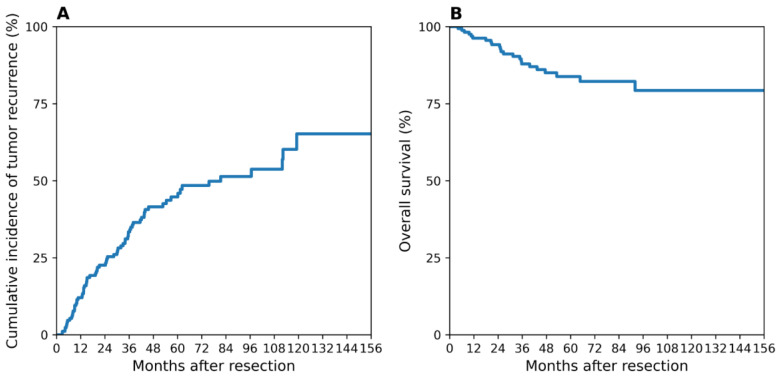
Cumulative incidence of tumor recurrence and survival rates. (**A**) 74 of 171 patients (68.2%) experienced tumor recurrence during the follow-up period; (**B**) Overall survival curve of the cohort. Twenty-three of 171 patients (79.3%) died during follow-up.

During a median follow-up of 4.4 ± 3.4 years (range, 3.2 months–15.5 years), 74 patients (68.2%) experienced tumor recurrence, and 23 patients (79.3%) died (Figure 1).

**Figure 2:**
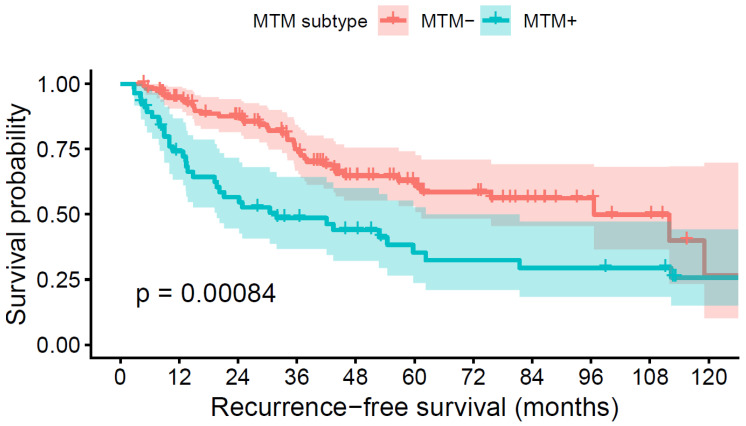
Recurrence-free survival according to MTM subtype. Kaplan–Meier curves demonstrate significantly lower recurrence-free survival in MTM-positive tumors compared with MTM-negative tumors (log-rank *p* = 0.00084).

Patients with MTM-positive tumors showed significantly worse recurrence-free survival compared with those with MTM-negative tumors (log-rank *p* = 0.00084), as illustrated in Figure 1. The separation of survival curves was evident early after surgery and persisted throughout long-term follow-up, reflecting the aggressive biological behavior associated with the MTM subtype (Figure 2).

In the Cox regression analysis, the macrotrabecular-massive (MTM) subtype was identified as an independent predictor of recurrence (HR = 1.881; 95% CI, 1.143–3.095; *p* = 0.013) (Table 2).

In contrast, lymphoid cell infiltration and tertiary lymphoid structures (TLS) did not demonstrate a significant association with recurrence or survival.

The Kaplan–Meier curves illustrate that the cumulative incidence of recurrence was significantly higher in the MTM group than in the non-MTM group (74.2% vs. 73.4%, *p* = 0.001).

**Figure 3:**
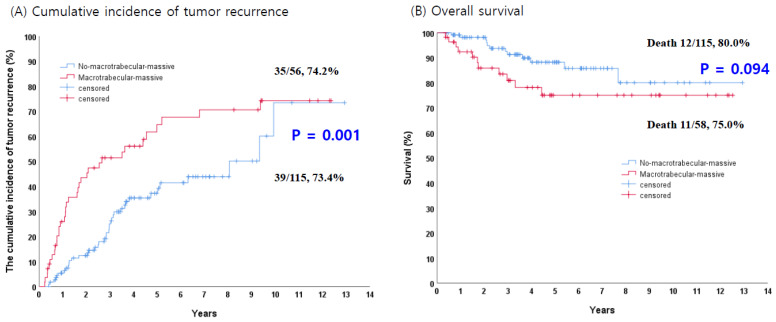
Recurrence patterns and overall survival according to MTM subtype. (**A**) Patients with MTM-positive tumors showed a significantly higher cumulative incidence of recurrence throughout the follow-up period compared with those with MTM-negative tumors. (**B**) Overall survival tended to be worse in the MTM-positive group compared with the MTM-negative group.

However, there was no significant difference in overall survival (OS) between the two groups (80.0% vs. 75.0%, *p* = 0.094) (Figure 3).

### 3.3. Early and Late Recurrence Patterns

Among the 74 patients who developed recurrence, 35 (47.3%) experienced early recurrence (<24 months) and 39 (52.7%) experienced late recurrence (≥24 months). As shown in Table 3, the proportion of MTM-positive tumors was significantly higher in the early recurrence group than in the late recurrence group (62.9% vs. 33.3%, *p* = 0.021). Patients with early recurrence also tended to exhibit higher preoperative tumor marker levels and a greater prevalence of microvascular invasion, although these differences did not consistently reach statistical significance. Other baseline characteristics were comparable between the two groups.

Definition: Recurrence timing was categorized as early (<24 months) or late (≥24 months) based on the interval between surgery and radiologic recurrence. Patients without recurrence were excluded from this comparison.

Statistics: Continuous variables are reported as mean ± SD and analyzed using Welch’s *t*-test. Categorical variables are reported as n (%) and analyzed using chi-square or Fisher’s exact test as appropriate.

**Figure 4:**
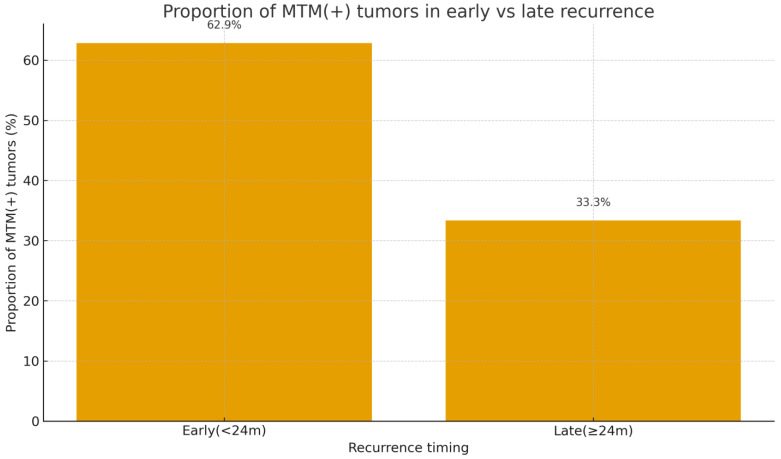
Proportion of MTM-positive tumors in early and late recurrence groups. MTM-positive tumors were more frequent in the early recurrence group than in the late recurrence group.

In univariate logistic regression (Table 4), MTM subtype was significantly associated with early recurrence (OR = 4.16, 95% CI = 1.38–12.49, *p* = 0.011). After adjustment for age, cirrhosis, microvascular invasion, AFP, and PIVKA-II, MTM subtype remained an independent predictor of early recurrence with borderline statistical significance (adjusted OR = 3.47, 95% CI = 0.88–13.59, *p* = 0.075). A forest plot summarizing the multivariable model is presented in Supplementary Figure S1. The distribution of MTM-positive tumors in early versus late recurrence groups is illustrated in Figure 4.

Model description: The multivariable logistic regression model was constructed using variables clinically associated with recurrence risk, including MTM subtype, age, cirrhosis, MVI, AFP, and PIVKA-II. Continuous variables (AFP and PIVKA-II) were log-transformed (log10) due to right-skewed distributions.

Definition: Early recurrence was defined as recurrence occurring within 24 months following curative surgical resection.

## 4. Discussion

In this study, we demonstrated that the macrotrabecular-massive (MTM) subtype of hepatocellular carcinoma (HCC) is associated with a significantly higher risk of recurrence following surgical resection, consistent with prior reports highlighting its aggressive clinical course and poor prognosis [17,20]. Several large cohort and multicenter studies have established MTM-HCC as a histologically distinct entity, accounting for approximately 10–20% of HCC cases, with strong correlations to vascular invasion, larger tumor burden, and elevated serum alpha-fetoprotein (AFP) levels [17,21,22]. These findings reinforce the clinical utility of recognizing MTM morphology for risk stratification in routine practice.

Recent work has further clarified the biological underpinnings of MTM-HCC. Transcriptomic and immunohistochemical analyses indicate enrichment of angiogenesis-related pathways, with marked upregulation of angiopoietin-2 (Ang2) and vascular endothelial growth factor A (VEGFA) [23]. Such alterations drive aberrant vascular remodeling and may contribute to the higher incidence of early recurrence and intrahepatic dissemination observed in these tumors. This molecular context not only explains the aggressive clinical phenotype but also provides a rationale for therapeutic targeting. Indeed, anti-angiogenic agents, either alone or in combination with immune checkpoint inhibitors, may have particular relevance for MTM-HCC patients, a hypothesis supported by the efficacy of VEGF/PD-L1 dual blockade in recent systemic therapy trials [24,25].

Radiologic advances have also opened new opportunities for non-invasive detection of MTM-HCC. Rhee et al. proposed gadoxetic acid–enhanced MRI criteria that identify MTM subtype based on arterial phase hypovascular components and ancillary features such as intratumoral arteries and non-smooth margins [15]. These imaging biomarkers demonstrated not only high diagnostic specificity but also prognostic significance, as patients fulfilling MRI-based MTM criteria exhibited inferior survival after surgery [15]. This highlights the potential role of imaging as a surrogate for tissue diagnosis, facilitating early risk stratification and treatment planning without the need for invasive biopsy.

Notably, despite the higher recurrence rate observed in MTM-positive tumors, overall survival remained comparable between groups. This apparent discrepancy may be explained by the impact of post-recurrence management, including locoregional or systemic salvage therapies, which can attenuate the effect of recurrence on long-term survival. Early recurrence after curative resection is widely recognized as a surrogate marker of aggressive tumor biology in HCC, reflecting microscopic residual disease, vascular invasion, and early intrahepatic dissemination. In the present study, the MTM subtype was markedly enriched in the early recurrence group, and MTM-positive tumors demonstrated a significantly higher likelihood of recurring within 24 months after surgery. This strong association suggests that MTM-HCC represents a biologically aggressive phenotype characterized by pro-angiogenic activity, increased cellular proliferation, and a higher propensity for vascular infiltration.

Importantly, MTM subtype remained a significant or borderline significant predictor of early recurrence even after adjusting for known risk factors, supporting its potential role as an independent histopathologic marker of early postoperative relapse. However, it should be acknowledged that MTM histology is strongly correlated with established adverse tumor features such as tumor size, microvascular invasion, and poor differentiation. Therefore, MTM may function as a surrogate marker reflecting aggressive tumor biology rather than a fully independent biological entity. These findings are consistent with previous reports describing MTM-HCC as a high-risk subtype associated with microvascular invasion, elevated tumor markers, and inferior oncologic outcomes. Although MTM-positive tumors were more frequently associated with early recurrence, the number of patients in the early recurrence subgroup was relatively small, and several associations reached only borderline statistical significance. Therefore, these findings should be interpreted cautiously and regarded as exploratory.

Clinically, the identification of MTM subtype at the time of resection may help stratify patients requiring intensified surveillance during the first two years after surgery, the period when early recurrence is most likely to occur and most prognostically impactful. Furthermore, patients with MTM-HCC may benefit from consideration of adjuvant therapies or more aggressive locoregional control strategies. Future studies integrating MTM histology with molecular profiling and immune microenvironmental features may provide a more refined understanding of its recurrence patterns and help guide individualized postoperative management.

From an immune perspective, our study did not find evidence that lymphoid infiltration or tertiary lymphoid structures (TLS) improved recurrence-free or overall survival. This stands in contrast to findings in other solid tumors, where tumor-infiltrating lymphocytes (TILs) consistently predict better outcomes [26,27]. In HCC, however, results have been heterogeneous. Some studies have reported that increased densities of CD8+ T cells, FOXP3+ regulatory T cells, or granzyme B+ lymphocytes are associated with prolonged survival [28], whereas others have found limited or no prognostic impact of generalized lymphoid infiltration [29]. This discrepancy may be explained by the unique immunosuppressive environment of the cirrhotic liver, where chronic inflammation, regulatory immune cell populations, and stromal signaling attenuate effective anti-tumor immunity [30,31]. Moreover, conventional histologic evaluation lacks the resolution to distinguish beneficial effector T-cell subsets from suppressive or exhausted populations, thereby masking clinically relevant associations [32]. Assessment of the immune microenvironment was limited by the use of hematoxylin and eosin staining alone, without immunohistochemical characterization of immune cell subsets or tertiary lymphoid structures, which may have masked clinically relevant associations.

Clinically, the recognition of MTM subtype carries important implications. Pathologists have demonstrated high reproducibility in diagnosing MTM features on both resection and biopsy specimens [17,21], and radiologists now have validated MRI-based tools for non-invasive identification [15]. These diagnostic advances allow clinicians to identify high-risk patients preoperatively, facilitating personalized management. Despite the poor prognosis associated with MTM, current international and regional guidelines, including those from the Korean Liver Cancer Association (KLCA-NCC), do not yet offer specific treatment algorithms tailored to this subtype [30]. However, given its consistent association with early recurrence and poor survival, MTM-HCC should be considered a candidate population for intensified surveillance and prioritized enrollment in adjuvant or neoadjuvant clinical trials exploring immunotherapy and targeted therapies [24,33].

Our study has several limitations. The retrospective, single-center design may limit generalizability, and our assessment of lymphoid infiltration was based solely on hematoxylin and eosin evaluation without advanced immunophenotyping. We also did not incorporate imaging biomarkers, such as the MRI-based criteria recently proposed, into our analysis. In addition, the long inclusion period may have introduced heterogeneity in surgical techniques, imaging quality, and postoperative surveillance strategies. Detailed information regarding recurrence patterns (intrahepatic versus extrahepatic, solitary versus multifocal) and post-recurrence treatment strategies was not uniformly available, which may have influenced survival outcomes.

Future studies should adopt prospective, multicenter designs and integrate histopathological, molecular, immunological, and imaging features to build comprehensive prognostic models. Such approaches may ultimately refine patient selection for adjuvant therapies and improve outcomes in this particularly high-risk subgroup of HCC.

## 5. Conclusions

The macrotrabecular-massive subtype is a robust predictor of recurrence in hepatocellular carcinoma following curative resection. In contrast, lymphoid infiltration and tertiary lymphoid structures did not confer prognostic benefit in this cohort. These results highlight the necessity of incorporating MTM subtype into risk assessment models and support the exploration of adjuvant therapeutic strategies tailored to this high-risk patient population.

## Figures and Tables

**Table 1:** Baseline characteristics of patients with hepatocellular carcinoma treated by surgical resection.

Variables	Recurrence N = 74	No-Recurrence N = 97	*p*-Value
Age, years	65.1 ± 8.6	61.6 ± 10.8	0.021
Male (%)	58 (78.4)	78 (80.4)	0.744
BMI, kg/m^2^	24.1 ± 3.0	24.3 ± 3.1	0.868
Etiology			0.691
HBV (%)	55 (74.3)	79 (81.4)	
HCV (%)	3 (4.1)	3 (3.1)	
Alcohol (%)	1 (1.4)	0 (0)	
NASH (%)	8 (10.8)	8 (8.2)	
Unknown (%)	7 (9.5)	7 (7.2)	
Diabetes (%)	14 (18.9)	23 (23.7)	0.451
Hypertension (%)	25 (33.8)	31 (32.0)	0.801
Liver cirrhosis (%)	35 (47.3)	49 (50.5)	0.677
AFP	2308 ± 1153	1727 ± 817	0.672
PIVKA-II	2233 ± 919	4681 ± 3194	0.536
Child–Pugh class			0.634
A (%)	73 (98.6)	94 (96.9)	
B (%)	1 (1.4)	3 (3.1)	
C (%)	0 (0)	0 (0)	
Maximal tumor size, cm	4.3 ± 3.2	3.4 ± 2.5	0.032
Tumor number	1.5 ± 1.6	1.4 ± 2.4	0.877
Resection margin positive (%)	2 (2.7)	3 (3.1)	1.000
Microvascular invasion (%)	29 (39.2)	34 (35.1)	0.578
Portal vein invasion (%)	8 (10.8)	8 (8.2)	0.604
Bile duct invasion (%)	2 (2.7)	3 (3.1)	
BCLC stage			0.230
Stage 0 (%)	14 (18.9)	28 (28.9)	
Stage A (%)	49 (66.2)	60 (61.9)	
Stage B (%)	11 (14.9)	9 (9.3)	
Stage C (%)	0 (0)	0 (0)	
Stage D (%)	0 (0)	0 (0)	
AJCC 8th stage			0.244
Stage IA (%)	13 (17.6)	29 (29.9)	
Stage IB (%)	28 (37.8)	38 (39.2)	
Stage II (%)	21 (28.4)	21 (21.6)	
Stage IIIA (%)	2 (2.7)	0 (0)	
Stage IIIB (%)	9 (12.2)	8 (8.2)	
Stage IVA (%)	1 (1.4)	1 (1.0)	
Stage IVB (%)	0 (0)	0 (0)	
Lymphoid cell infiltration positive (%)	59 (79.7)	85 (87.6)	0.160
Tertiary lymphoid structure positive (%)	2 (2.7)	7 (7.2)	0.302
Tumor infiltrating lymphocyte positive (%)	46 (62.2)	67 (69.1)	0.344
Macrotrabecular-massive subtype (%)	35 (47.3)	21 (21.6)	<0.001
Edmonson grade			0.698
0 (%)	0 (0)	1 (1.0)	
1 (%)	0 (0)	1 (1.0)	
2 (%)	19 (25.7)	24 (24.7)	
3 (%)	45 (60.8)	61 (62.9)	
4 (%)	9 (12.2)	10 (10.3)	
5 (%)	1 (1.4)	0 (0)	
Follow-up duration, years	4.2 ± 2.9	4.5 ± 3.2	0.527

Note: Data are expressed as mean ± standard deviation or number (percentage). *p*-values were calculated using the χ^2^ test or Student’s *t*-test, as appropriate. HBV, hepatitis B virus; HCV, hepatitis C virus; NASH, nonalcoholic steatohepatitis; AFP, alpha-fetoprotein; PIVKA-II, protein induced by vitamin K absence-II.

**Table 2:** Baseline factors associated with the recurrence of hepatocellular carcinoma in Cox regression analysis.

Variables	Univariate Analysis		Multivariate Analysis	
	HR (95% CI)	*p*-Value	HR (95% CI)	*p*-Value
Age, years	1.036 (1.004–1.070)	0.028	1.016 (0.992–1.041)	0.195
Liver cirrhosis	1.137 (0.621–2.084)	0.677	1.237 (0.748–2.046)	0.407
Maximal tumor size, cm	1.132 (1.011–1.267)	0.032	1.080 (0.996–1.175)	0.062
Resection margin positive	1.149 (0.187–7.058)	0.881		
Microvascular invasion	1.194 (0.639–2.233)	0.579	1.290 (0.768–2.167)	0.337
Portal vein invasion	1.348 (0.481–3.779)	0.570		
Lymphoid cell infiltration positive	0.555 (0.242–1.272)	0.164		
Tertiary lymphoid structure positive	0.357 (0.072–1.771)	0.208		
Tumor infiltrating lymphocyte positive	0.736 (0.389–1.391)	0.345		
Macrotrabecular-massive subtype	3.248 (1.671–6.313)	0.001	1.881 (1.143–3.095)	0.013

Note: HR, hazard ratio; CI, confidence interval. Multivariate analysis included variables with *p* < 0.1 in the univariate analysis.

**Table 3:** Comparison of clinicopathologic characteristics between early and late recurrence groups.

Variable	Early (<24 m)	Late (≥24 m)	*p*-Value
age, years	63.5 ± 9.1	66.4 ± 8.0	0.157
AFP, ng/mL	2300.6 ± 9647.7	2316.0 ± 9779.6	0.995
PIVKA-II, mAU/mL	4478.4 ± 9865.0	278.0 ± 452.0	0.036
MTM+	22/35 (62.9%)	13/39 (33.3%)	0.021
MTM−	13/35 (37.1%)	26/39 (66.7%)	0.021
MVI (+), %	19/35 (54.3%)	10/39 (25.6%)	0.023
Cirrhosis (+), %	22/35 (62.9%)	15/39 (38.5%)	0.063
Male, %	26/35 (74.3%)	32/39 (82.1%)	0.598

Abbreviations: MTM, macrotrabecular-massive subtype; AFP, α-fetoprotein; PIVKA-II, protein induced by vitamin K absence-II; MVI, microvascular invasion.

**Table 4:** Logistic regression analysis identifying clinicopathologic factors associated with early recurrence (<24 months).

Variables	OR	95% CI	*p*-Value
MTM (+) vs. MTM (−)	3.47	0.88–13.59	0.075
MVI (+ vs. −)	2.71	0.72–10.24	0.141
Cirrhosis (yes vs. no)	2.23	0.66–7.53	0.198
Age (per year)	0.97	0.90–1.05	0.488
log10 (AFP)	0.71	0.36–1.39	0.316
log10 (PIVKA-II)	1.55	0.72–3.34	0.262

Abbreviations: OR, odds ratio; CI, confidence interval; AFP, α-fetoprotein; PIVKA-II, protein induced by vitamin K absence-II; MVI, microvascular invasion; MTM, macrotrabecular-massive subtype.

## Data Availability

The data used to support the findings of this study are available from the corresponding author upon request.

## Supplementary Materials

The following supporting information can be downloaded at: https://www.mdpi.com/article/10.3390/jcm15020502/s1, Figure S1: Multivariable logistic regression for predictors of early recurrence. Forest plot showing odds ratios and 95% confidence intervals for factors associated with early recurrence (<24 months).

## Abbreviations

AFP, alpha-fetoprotein; AJCC, American Joint Committee on Cancer; Ang2, angiopoietin-2; APC, article processing charge; BCLC, Barcelona Clinic Liver Cancer; BMI, body mass index; CI, confidence interval; CT, computed tomography; HBV, hepatitis B virus; HCC, hepatocellular carcinoma; HCV, hepatitis C virus; HR, hazard ratio; IF, impact factor; IHC, immunohistochemistry; IRB, Institutional Review Board; MRI, magnetic resonance imaging; MTM, macrotrabecular-massive; OS, overall survival; PIVKA-II, protein induced by vitamin K absence-II; RFA, radiofrequency ablation; RFS, recurrence-free survival; SPSS, Statistical Package for the Social Sciences; TLS, tertiary lymphoid structure; TIL, tumor-infiltrating lymphocyte; VEGFA, vascular endothelial growth factor A; KLCA-NCC, Korean Liver Cancer Association–National Cancer Center.
