# Supplementary figures and images for: Macrotrabecular-Massive Subtype Is Associated with a High Risk of the Recurrence of Hepatocellular Carcinoma
Source: J Clin Med. 2026 Jan 8;15(2):502. doi: 10.3390/jcm15020502 (PMC12841605; doi:10.3390/jcm15020502)

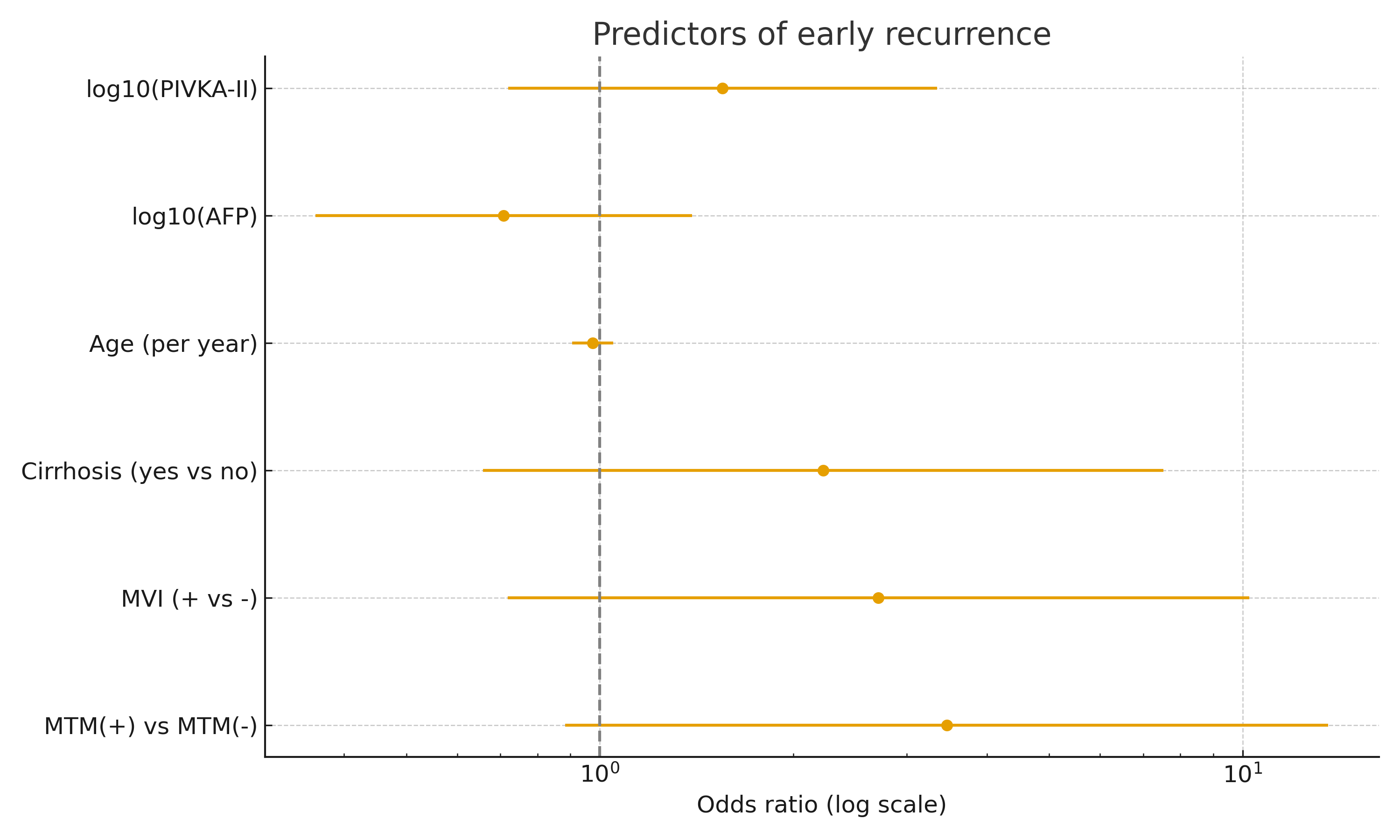

Supplement: Supplementary file 1 [file jcm-15-00502-s001.zip › jcm-4004650-supplementary.png]
